# Unveiling Timetable for Physical Therapy after Single-Level Lumbar Surgery for Degenerative Disc Disease: Insights from a Systematic Review and Meta-Analysis

**DOI:** 10.3390/jcm13092553

**Published:** 2024-04-26

**Authors:** Alberto Ruffilli, Marco Manzetti, Alessandro Cargeli, Giovanni Viroli, Marco Ialuna, Matteo Traversari, Fabio Vita, Isabella Sofia Giannini, Cesare Faldini

**Affiliations:** 1 1st Orthopaedic and Traumatologic Clinic, IRCCS Istituto Ortopedico Rizzoli, 40136 Bologna, Italy; alberto.ruffilli@ior.it (A.R.); alessandro.cargeli@ior.it (A.C.); giovanni.viroli@ior.it (G.V.); marco.ialuna@ior.it (M.I.); matteo.traversari@ior.it (M.T.); fabio.vita@ior.it (F.V.); isabellasofia.giannini@ior.it (I.S.G.); cesare.faldini@ior.it (C.F.); 2 Department of Biomedical and Neuromotor Science—DIBINEM, University of Bologna, 40126 Bologna, Italy

**Keywords:** early physical therapy, lumbar disc disease, timing, outcomes, complications

## Abstract

**Background:** Postoperative physical therapy emerges as a pivotal element of the rehabilitation process, aimed at enhancing functional recovery, managing pain, and mitigating the risk of further complications. The debate concerning the optimal timing of physical therapy intervention post-surgery remains unresolved; in particular, whether to initiate physical therapy immediately or to wait weeks is of particular interest. The aim of this study is to review the available literature regarding the optimal timing of physical therapy initiation and the outcomes obtained. **Methods:** This review was carried out in accordance with the Preferential Reporting Items for Systematic Reviews and Meta-analysis (PRISMA) guidelines. This search was carried out in February 2024. Only peer-reviewed articles were considered for inclusion. **Results:** Fourteen studies were included. The primary outcomes assessed in the included studies were the following: 12-week and 12-month low back pain, return to work, function and disability, psychological status, patient satisfaction, and complications associated with early physical therapy. A meta-analysis was performed concerning low back pain after lumbar discectomy at 12 weeks and 12 months and complications after early physical therapy after lumbar discectomy and lumbar interbody fusion. A significant difference was found between early and standard physical therapy in terms of low back pain at 12–18 months (*p* = 0.0062); no significant differences were found in terms of complications, both for discectomy and arthrodesis. **Conclusions:** This review indicates that employing early rehabilitation strategies for intervertebral disc disease could enhance results in terms of pain and disability without an enhanced risk of complications.

## 1. Introduction

The ongoing demographic transformation towards a more aged global population is set to profoundly reshape healthcare priorities, particularly in the realm of managing degenerative spine diseases [1]. The World Health Organization’s projection states that by 2030, one in every six individuals worldwide will be over the age of 60, bringing to the forefront the urgent need to address the burgeoning health challenges associated with this shift [2].

Among these challenges, degenerative conditions such as lumbar disc herniation (LDH), degenerative scoliosis, and lumbar stenosis are increasingly prevalent, driven by factors including aging, lifestyle, and genetic predisposition. These conditions, characterized by the progressive degeneration of spinal structures, significantly impact patients’ quality of life, often necessitating surgical treatment for relief [3,4,5].

Postoperative physical therapy emerges as a pivotal element of the rehabilitation process, aimed at enhancing functional recovery, managing pain, and mitigating the risk of further complications [6,7]. However, the debate concerning the optimal timing of physical therapy intervention post-surgery remains unresolved; in particular, whether to initiate physical therapy immediately or to wait weeks is of particular interest. Contemporary research supports initiating physical therapy within two weeks postoperatively for lumbar discectomy [8] and between three and six weeks postoperatively for single-level lumbar interbody fusion [9]. This timing has been shown to effectively reduce postoperative low back pain, enhance functional recovery, and decrease disability. Early physical therapy should be integrated into multimodal Enhanced Recovery After Surgery (ERAS) programs, which have been shown through empirical evidence to significantly improve outcomes. These programs not only relieve postoperative pain and speed up functional recovery but also have the added benefit of increasing patient turnover in hospitals. By shortening hospital stays, early physical therapy allows for a quicker return to daily activities and work, thus offering distinct advantages over traditional rehabilitation protocols. Hospital managers should investigate the importance of early physical therapy to further enhance the efficiency of patient management and support faster recovery processes [10]. 

This review endeavors to synthesize the existing literature, focusing on the timing of physical therapy initiation and the outcomes obtained. A comprehensive review of the literature was conducted to determine the optimal timing for initiating physical therapy following surgery. This includes an analysis of early vs. delayed initiation of physical therapy interventions with the aim of understanding their impact on patient outcomes such as functional recovery, pain management, and complications. 

The goal is to identify which timing of physical therapy initiation is most effective in improving functional capacity, alleviating pain, and facilitating a return to daily activities, while also considering the potential risks of initiating therapy too early. By addressing these two critical aspects, this review aims to furnish clinical practice by identifying the most effective strategies for physical therapy intervention post-surgery for degenerative intervertebral disc disease and ensuring that recommendations are tailored to meet the individual needs of patients.

## 2. Materials and Methods

### 2.1. Review Design

A systematic review of the literature regarding the optimal timing of postoperative physical therapy after spine surgery for degenerative disc disease was carried out following the Preferred Reporting Items for Systematic Reviews and Meta-Analyses (PRISMA) guidelines [11]. The Oxford Centre for Evidence-Based Medicine (OCEBM) [12] was used to assess the level of evidence in the included studies (full version for randomized and non-randomized clinical trials, modified version for all other studies).

The considered inclusion criteria were the following: papers describing the optimal timing of postoperative physical therapy after elective lumbar spine procedures in degenerative intervertebral disc disease, either discectomy or one-level lumbar interbody fusion. 

The applied exclusion criteria were the following: Isolated case reports/series with less than 5 patients, technical notes, expert opinions, literature reviews, meta-analyses, and biomechanical and/or in vitro studies; papers providing incomplete data or not providing data regarding optimal timing for postoperative physical therapy; papers describing outcomes in cervical spine, tumoral or metastatic spine, trauma, infection, and revision surgery. Papers reporting results in patients with a diagnosis of rheumatologic disease, connective tissue disease, or malabsorptive disorder were also excluded because these conditions can affect bone and muscle quality, confounding the results. Studies not indicating the diagnosis but excluding patients undergoing cervical or thoracic spine, tumoral or metastatic spine, trauma, and revision surgery were also included. Articles in English in peer-reviewed journals that met the population, intervention, comparison, and outcome criteria on systematic reviews were considered for inclusion. Randomized controlled trials, prospective and retrospective cohort studies, and case series (CS) were considered for inclusion.

### 2.2. Search Strategy

An electronic systematic search of the available English literature on three large electronic databases (Pubmed-MEDLINE, Scopus, and Google Scholar) was performed over the years 1994–2024 to identify eligible studies. The online literature search was conducted in February 2024 by two authors. The authors stated the following research questions: “Is there an optimal time for starting physical therapy after degenerative disc disease surgery?”; “Is early physical therapy beneficial when compared to standard physical therapy?”; and “Is early physical therapy safe, or does it bring more complications when compared to standard physical therapy?”.

The search was conducted using combinations of the following keywords: “lumbar disc disease”, “discectomy”, “exercise therapy”, “rehabilitation”, “intervertebral disc degeneration”, “lumbar spine fusion”, “physical therapy”, “lumbar pain”, “lumbar physical therapy”, “visual analogue scale”, “timing”, “low back pain”, “optimal timing”, “early physical therapy”, “ODI”, “outcomes”, “complications”, “exercises”, “pain”, and “disability”. 

### 2.3. Study Selection

**Figure 1:**
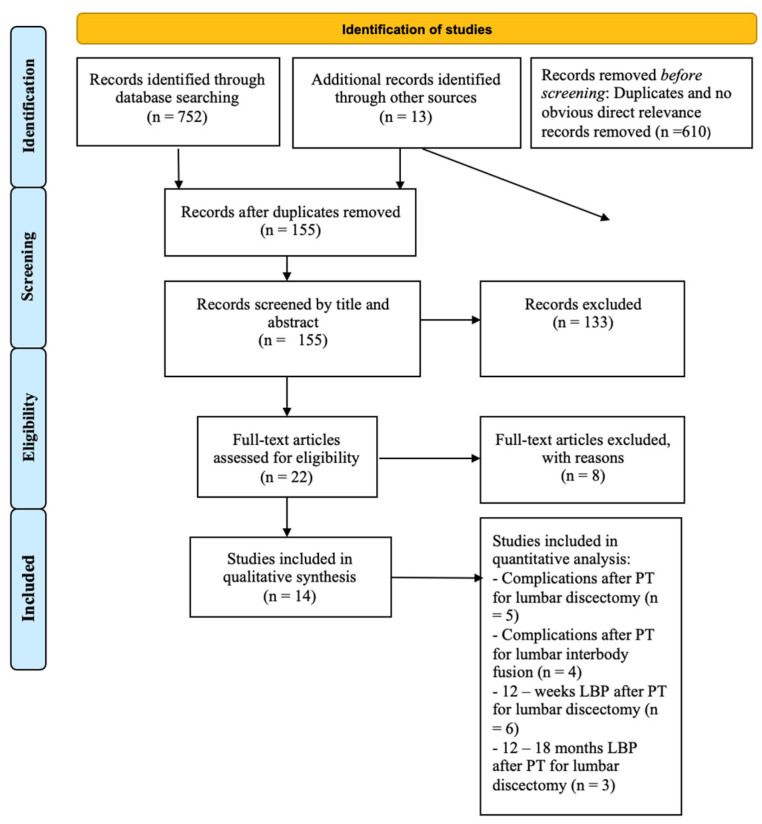
Prisma 2009 flow diagram of the included studies; LBP = low back pain; PT = physical therapy.

After screening the titles and abstracts, the full-text articles were obtained and reviewed. A manual search of the bibliography of each of the relevant articles was also performed to identify potentially missed eligible papers. Reviews and meta-analyses were also analyzed to potentially broaden the search for studies that might have been missed through the electronic search. Duplicates were removed. The study selection process was carried out in accordance with the PRISMA flowchart [11] (Figure 1). 

The present systematic review was accepted for registration in the PROSPERO database for systematic reviews [13] (ID: CRD42024523304). Ethical approval and institutional review board approval were not required because this study retrieved and synthesized data from already published studies.

### 2.4. Data Extraction

Two authors extracted the data through a standardized data collection form. Three authors checked the data for accuracy, and inconsistent results were discussed. Data concerning study design, number and demographics of patients, surgical procedure, timing of physical therapy, intervention type, control, results, and complications were extracted and summarized in Table 1. 

Post-physical-therapy pain, disability, function, return to work, patient satisfaction, and complications at different timings of physical therapy initiation were considered as outcome measures. When studies involved patients with early timing of postoperative physical therapy not solely limited to degenerative disc disease patients (such as tumors or fractures), data about patients with degenerative disc disease were pooled; if this was not possible, the study was excluded.

### 2.5. Methodological Quality Assessment of Included Studies

**Figure 2:**
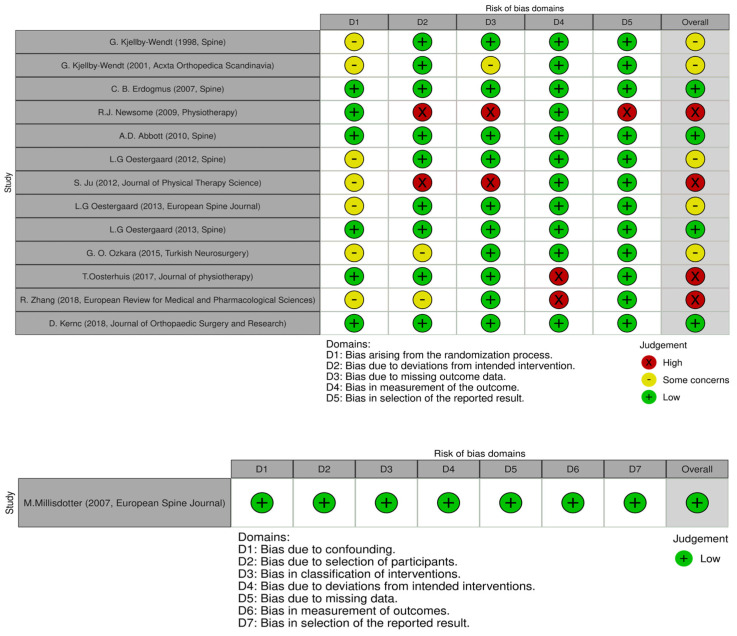
ROB 2.0 and ROBINS-I tools used for the included studies [8,9,14,15,16,17,18,19,20,21,22,23,24,25].

**Figure 3:**
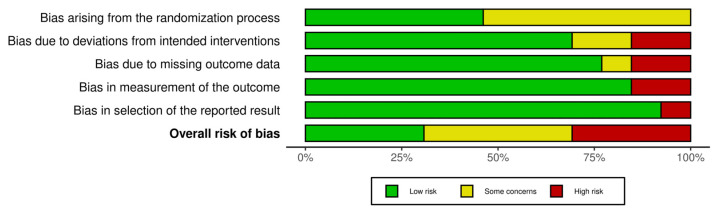
ROB2 tool plot summary.

The Revised Cochrane Risk of Bias tool for randomized trials (RoB 2.0) [14] was used to assess the methodological quality of the included studies. The quality of each study was reported by assessing 5 domains: randomization process, deviation from intended interventions, missing outcomes, measurement of the outcomes, and selection of the reported results. Each domain can present a low, some-concern, or high risk of bias; these combined together form an overall risk of bias. For each included study, the total risk of bias was categorized as low risk with 5 low-risk domains, some-concern risk with at least one some-concern domain, and high risk with ≥1 high-risk domain or multiple some-concern domains. A similar tool, the Risk of Bias in Non-Randomized Studies of Interventions (ROBINS-I) tool, was used for evaluating the methodological quality of only the non-randomized studies included [8]. As with the evaluation of titles and abstracts, any disagreement was solved by a senior author. Details on the quality of the studies included are summarized in Figure 2 and Figure 3.

### 2.6. Statistical Analysis

Meta-analyses were performed when at least three studies were comparable. This was possible for complications after physical therapy for lumbar discectomy and interbody lumbar fusion, for 1-year residual back pain after physical therapy for lumbar discectomy, and for 12-week residual back pain after physical therapy for lumbar discectomy.

For complication rates, dichotomous models were evaluated; for post-physical therapy back pain, a standardized mean difference evaluation was performed. The analysis was carried out using the log odds ratio with 95% CI and *p* value as the outcome measure of effect size. A random-effects model was fitted to the data. The amount of heterogeneity (i.e., tau^2^) was estimated using the restricted maximum likelihood estimator. In addition to the estimate of tau^2^, the Q-test for heterogeneity and the I^2^ statistic were reported. In cases where any amount of heterogeneity was detected (i.e., tau^2^ > 0, regardless of the results of the Q-test), a prediction interval for the true outcomes was also provided. Studentized residuals and Cook’s distances were used to examine whether studies may be outliers and/or influential in the context of the model. Studies with a studentized residual larger than the 100 × (1 − 0.05/(2 × k))th percentile of a standard normal distribution were considered potential outliers (i.e., using a Bonferroni correction with two-sided alpha = 0.05 for k studies included in the meta-analysis). Studies with a Cook’s distance larger than the median plus six times the interquartile range of the Cook’s distances were considered to be influential. The rank correlation test and the regression test, using the standard error of the observed outcomes as a predictor, were used to check for funnel plot asymmetry. All statistical analyses were conducted with Jamovi version 2.2 (The Jamovi Project, Sydney, Australia) software. A *p* value < 0.05 was considered to be significant.

## 3. Results

### 3.1. Included Studies and Population

Initially, a total of 752 studies were found through an electronic search. Before title screening, 610 articles were excluded after duplication removal or for having no obvious direct relevance to the topic. A total of 155 records were screened by title and abstract, leading to the exclusion of 133 records. After screening, 22 studies were assessed for eligibility. The inclusion criteria were not met by eight studies, such as those that included revision surgery patients; those with traumatic, neoplastic, or cervical spine diseases; or those that reported no or insufficient postoperative physical therapy timing data or surgical procedure data.

Eventually, 14 studies [8,9,15,16,17,18,19,20,21,22,23,24,25,26] met the inclusion criteria and were included in this systematic review for qualitative synthesis. Five different studies [8,15,21,23,26] were considered for quantitative analysis regarding complications after physical therapy for lumbar discectomy, four [9,16,19,24] for complications after physical therapy for lumbar interbody fusion, six [8,15,21,22,25,26] for 12-week residual back pain after physical therapy for lumbar discectomy, and three studies [8,15,21] for 1-year residual back pain after physical therapy for lumbar discectomy (Figure 1).

All of the included studies were designed in a randomized control trial fashion, except for one, which utilized prospectively collected data and enrolled patients using single-institution databases from various years of recruitment. The included studies reported data on a total of 993 patients (596 females, 60%), and the median age at surgery ranged from 32.5 ± 7.3 to 61.1 ± 8 years. In all the analyzed studies, each group of early physical therapy patients in which outcomes were evaluated was matched with a relatively homogeneous group composed of non-early physical therapy patients in which outcomes were evaluated. The included studies analyzed both small- and large-sized populations and were heterogeneous in the description of complications after physical therapy (Table 1).

### 3.2. Risk of Bias Assessment

Two authors assessed the risk of bias for each study using the ROB 2.0 and ROBINS-I tools; the results are shown in Figure 2. The studies indicated an overall risk of bias that was categorized as low, presenting some concerns, or high (respectively, 35.7%, 35.7%, and 28.6%). Four studies [18,22,23,26] had a high risk of bias due to the bias caused by the randomization process, deviations from the intended interventions, measurement of the outcomes, and selection of the reported results. Since most studies described outcome measurement well with clear definitions of the results, accurate and reliable outcome measurements, and low missing outcomes, they demonstrated low measurement outcome and low missing outcome data and low bias in the selection of the reported result items (<80%). Furthermore, the randomization process item was consistently moderate for most studies (~50%) since the randomization process may be skewed by the described procedure for randomization (Figure 3).

### 3.3. Timing of Physical Therapy

All the included studies evaluated the timing of the initiation of postoperative rehabilitation in their populations. Six distinct timings of early physical therapy (2 h, 1 day, 1 week, and 2 weeks postoperatively for lumbar discectomy; and 3 and 6 weeks postoperatively for lumbar interbody fusion) were discussed in this review.

Kjellby and colleagues [15] randomized two groups of patients who started an early active training program (EAT) at either postoperative day 1 or week 6 after a lumbar microdiscectomy. The EAT group patients had significantly less intense pain compared to the control group patients at 6 and 12 weeks after surgery. The range of motion of the lumbar spine was significantly more increased in the EAT group at 12 weeks after surgery compared to the controls. However, this trend was not replicated one year after surgery.

Erdogmus et al. [21] confirmed the importance of early physical therapy, starting from postoperative week 1 after lumbar discectomy. In this prospective randomized study, the authors randomized 120 patients into three groups of interventions (early physical therapy, sham therapy, and no treatment) and compared the results at 12 weeks and 1.5 years. There was a significant difference in low back pain at 12 weeks in early physical therapy patients compared to the untreated patients, which was not replicated at 1.5 years. These results are in contrast with those obtained by other studies [23,26], where early physical therapy was neither more effective nor more cost-effective than no referral.

Oestergaard et al. [9] performed a randomization of two groups of patients who had undergone lumbar fusion to initiate physical therapy early, at 6 weeks, or standard, at 12 weeks postoperatively. An early start of rehabilitation (6 weeks vs. 12 weeks) resulted in inferior outcomes; moreover, the improvement in the 12-week group was four times better compared to the 6-week group. 

These results are in contrast with those obtained by Abbott et al. [24], where early physical therapy at postoperative week 3 improved pain, functional disability, self-efficacy, outcome expectancy, and fear of movement when compared with no physical therapy for the first 3 postoperative weeks.

In another study [19], the randomization between early physical therapy at 3 weeks and a control group with no physical therapy for 3 months showed that early initiation of postoperative rehabilitation had better results in terms of muscle strength and walking speed. 

### 3.4. Outcomes Evaluated

This systematic review examined the primary outcomes assessed in the included studies: 12-week and 12-month low back pain, return to work, function and disability, psychological status, patient satisfaction, and complications associated with early physical therapy.

The intensity of low back pain at 12 weeks and 12 months following physical therapy, both post-lumbar interbody fusion and post-lumbar discectomy, has been analyzed. Merely two studies [9,24] specifically examined low back pain following physical therapy for lumbar interbody fusion, yielding contradictory results. 

Abbott et al. [24], in their randomized control trial, demonstrated that early postoperative rehabilitation can be safely administered after lumbar fusion, significantly reducing the VAS score for low back pain in comparison to control subjects at 3, 6, and 12 months, with no further differences noted at 2–3 years. 

These findings starkly contrast with those of another study [9], in which, at a 12-month follow-up, the early physical therapy group exhibited outcomes four times worse in terms of back pain when compared to the control group, suggesting that an early initiation of physical therapy may adversely affect overall outcomes.

When comparing early physical therapy with sham or no physical therapy in patients who have undergone lumbar discectomy, there is reasonable evidence that early physical therapy reduces pain at 12 weeks, as stated by the included studies.

However, a meta-analysis was performed on studies in which data regarding patients with low back pain after early or standard physical therapy could be pooled. This was possible for six studies that quantified low back pain at 12 weeks postoperatively [8,15,21,22,25,26] and for three studies that quantified low back pain at 12–18 months [8,15,21] postoperatively after lumbar discectomy.

**Figure 4:**
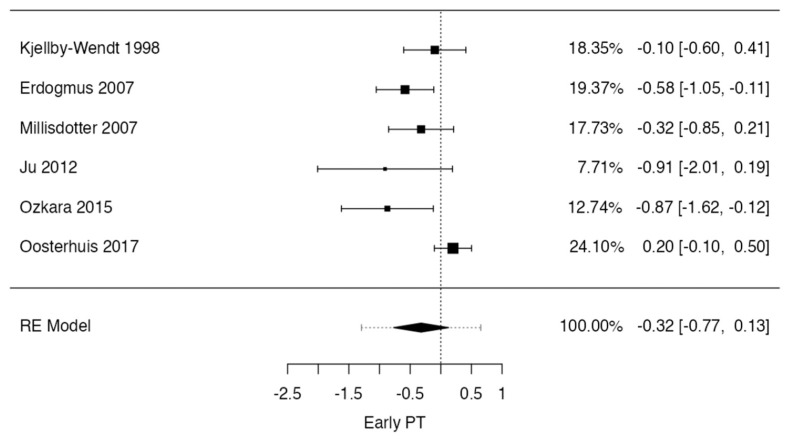
Forrest plot representation of the meta-analysis for low back pain at 12–week follow-up after lumbar discectomy [8,14,16,19,22,23].

In the low back pain at 12 weeks case, the observed standardized mean differences ranged from −0.9092 to 0.1991, with most estimates being negative (83%). The estimated average standardized mean difference based on the random-effects model was −0.3210 (95% CI: −0.7706 to 0.1287). Therefore, the average outcome did not differ significantly from zero (t (5) = −1.8349, *p* = 0.1260). According to the Q-test, the true outcomes appeared to be heterogeneous (Q (5) = 14.1591, *p* = 0.0146, tau^2^ = 0.1129, I^2^ = 61.9562%). A 95% prediction interval for the true outcomes was given as −1.2948 to 0.6529. Hence, although the average outcome was estimated to be negative, in some studies, the true outcome may in fact be positive. An examination of the studentized residuals revealed that one study [26] had a value larger than ± 2.6383 and may be a potential outlier in the context of this model. According to Cook’s distances, none of the studies could be overly influential. The regression test indicated funnel plot asymmetry (*p* = 0.0410), but not the rank correlation test (*p* = 0.4694) (Figure 4).

**Figure 5:**
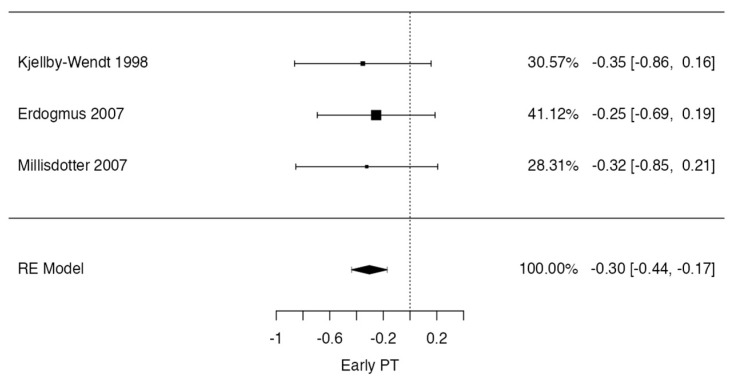
Forrest plot representation of the meta-analysis for low back pain at 12–18-month follow-up after lumbar discectomy [8,14,16].

In the low back pain at 12–18 months case, the observed standardized mean differences ranged from −0.3529 to −0.2531, with the majority of estimates being negative (100%). The estimated average standardized mean difference based on the random-effects model was −0.3036 (95% CI: −0.4364 to −0.1709). Therefore, the average outcome differed significantly from zero (t(2) = −9.8411, *p* = 0.0102). According to the Q-test, there was no significant amount of heterogeneity in the true outcomes (Q(2) = 0.0919, *p* = 0.9551, tau^2^ = 0.0000, I^2^ = 0.0000%). An examination of the studentized residuals revealed that one [21] of the studies had a value larger than ± 2.3940 and may be a potential outlier of this model. According to Cook’s distances, none of the studies could be considered to be overly influential. Neither the rank correlation nor the regression test indicated any funnel plot asymmetry (*p* = 1.0000 and *p* = 0.2881, respectively) (Figure 5).

Five trials [9,15,21,23,25] evaluated return-to-work differences between early physical therapy and controls. All trails, except for one [23], agree on the non-significant influence of early physical therapy on return to work. In one trial, more than 80% of participants returned to work after 1.5 years without any significant difference between groups [15]. On the other hand, Newsome and colleagues [23] reported that patients in the early physical therapy group showed a significantly more rapid return to work when compared to the control group. Unfortunately, a meta-analysis could not be conducted due to the impossibility of pooling data resulting from the heterogeneity in the timing of the return-to-work evaluation or due to missing data.

All studies included in this review, except for two [18,20], reported outcomes related to either functionality or disability following early physical therapy. The current literature trend suggests that there is evidence of significant differences in disability and function among patients who commence physical therapy early. Nevertheless, these significant differences become less pronounced when examining studies in which patients underwent single-level lumbar fusion. Indeed, Oestergaard and colleagues [9,16,17] have described in their investigations that early physical therapy does not outperform standard physical therapy regarding disability and function. Rather, it may demonstrate inferior results at a one-year follow-up when compared to standard physical therapy. These results are in contrast with those obtained by Abbott et al. [24], where patients who underwent early physical therapy treatment at 3 weeks significantly improved their disability and function scores when compared to the controls. Despite this, a meta-analysis could not be conducted due to the impossibility of pooling data. This was the result of either the heterogeneity in the timing of function and disability evaluations and the tools used for these evaluations or due to missing data.

Three trials reported on patient satisfaction [15,21,25] and four on the psychological status after early physical therapy [20,24,25,26]. Regarding patient satisfaction, one study [15] indicated that there was no significant difference in treatment outcome satisfaction among participants across different groups at any evaluated time point. In contrast, a separate investigation [21] conducted a follow-up two years post-surgery, revealing that 88% of the individuals who had undergone early, comprehensive physical therapy expressed satisfaction with their functional outcomes. This is in comparison to 67% of the participants in the control group reporting similar satisfaction levels.

The psychological well-being was meticulously delineated by Kjelby et al. in their randomized controlled trial [20], wherein patients who underwent early physical therapy showed significant improvement in anxiety levels and pain coping in their daily activities when compared with the controls. This finding received corroboration through the studies conducted by Abbott [24] and Ozakara [25]. Despite this, a meta-analysis could not be conducted due to the impossibility of pooling data. This was the result of either the heterogeneity in the timing of patient satisfaction and psychological status evaluations and the tools used for these evaluations or due to missing data.

When comparing early physical therapy with sham or no physical therapy, there is reasonable evidence to suggest that early physical therapy is safe and does not increase the risk of complications, as stated by the included studies. Four trials [9,17,24] reported complications in patients who underwent lumbar interbody fusion. Conversely, five trials [8,15,16,17,23] reported complications in patients who underwent lumbar discectomy. The adverse events reported included re-herniation, reoperation at a different or the same level, pseudarthrosis, loosening of the implant, and revision surgery. A meta-analysis was performed on studies in which data regarding patients with complications after early or standard physical therapy could be pooled; this was possible for four trials [9,17,24] that reported complications in patients who underwent lumbar interbody fusion and for five trials [8,20,21,23,26] that reported complications in patients who underwent lumbar discectomy.

**Figure 6:**
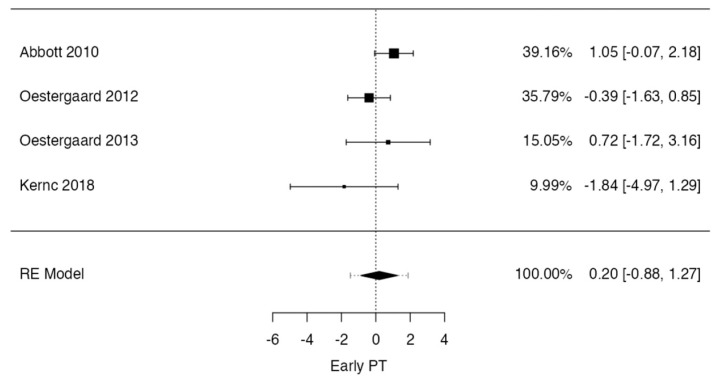
Forrest plot representation of the meta–analysis for complications after lumbar interbody fusion and physical therapy [9,18,20,24].

In the lumbar interbody fusion case, the observed log odds ratios ranged from −1.8412 to 1.0537, with the majority of estimates being negative (50%). The estimated average log odds ratio based on the random-effects model was 0.1959 (95% CI: −0.8756 to 1.2675). Therefore, the average outcome did not differ significantly from zero (z = 0.3584, *p* = 0.7201). According to the Q-test, there was no significant amount of heterogeneity in the true outcomes (Q(3) = 4.8407, *p* = 0.1838, tau^2^ = 0.4351, I^2^ = 37.4998%). A 95% prediction interval for the true outcomes was given as −1.4832 to 1.8751. Hence, although the average outcome was estimated to be positive, in some studies, the true outcome may in fact be negative. An examination of the studentized residuals revealed that none of the studies had a value larger than ± 2.4977, and hence there was no indication of outliers in the context of this model. According to Cook’s distances, none of the studies could be considered to be overly influential. Neither the rank correlation nor the regression test indicated any funnel plot asymmetry (*p* = 0.7500 and *p* = 0.3939, respectively) (Figure 6).

**Figure 7:**
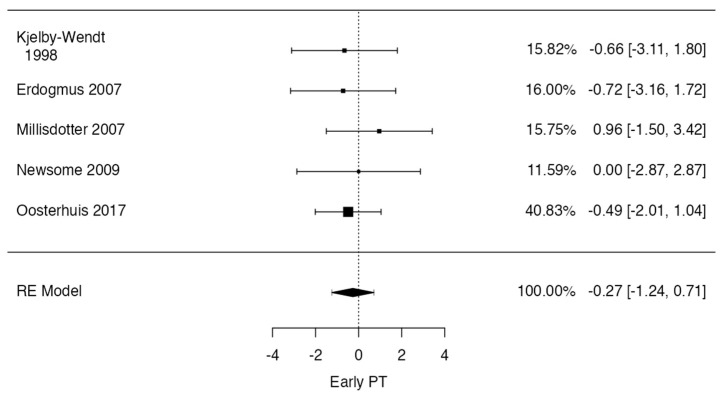
Forrest plot representation of the meta–analysis for complications after lumbar discectomy and physical therapy [8,14,16,17,23].

In the lumbar discectomy case, the observed log odds ratios ranged from −0.7191 to 0.9589, with the majority of estimates being negative (60%). The estimated average log odds ratio based on the random-effects model was −0.2665 (95% CI: −1.2433 to 0.7103). Therefore, the average outcome did not differ significantly from zero (z = −0.5348, *p* = 0.5928). According to the Q-test, there was no significant amount of heterogeneity in the true outcomes (Q(4) = 1.2941, *p* = 0.8624, tau^2^ = 0.0000, I^2^ = 0.0000%). An examination of the studentized residuals revealed that none of the studies had a value larger than ±2.5758, and hence there was no indication of outliers in the context of this model. According to Cook’s distances, none of the studies could be considered to be overly influential. Neither the rank correlation nor the regression test indicated any funnel plot asymmetry (*p* = 0.2333 and *p* = 0.7005, respectively) (Figure 7).

## 4. Discussion

Postoperative rehabilitation encompasses a diverse assortment of practices across different contexts. For certain practitioners, it consists merely in distributing a brochure accompanied by patient education to engage in walking. In contrast, European institutions frequently adopt a “Back Café” methodology [27], integrating psychosocial support alongside physical exercises. Alternatively, some propose a rigorous regimen of isometric strengthening exercises [18,19,22]. This work has analyzed the limited literature specifically dedicated to rehabilitation subsequent to lumbar discectomy or spinal fusion for intervertebral disc disease, seeking to determine the outcomes of early physical therapy after surgery for intervertebral disc disease. The timing for the optimal initiation of physical therapy was limited to a maximum of 4 weeks postoperatively for lumbar discectomy and 6 weeks for lumbar interbody fusion, obtaining moderate-quality evidence that comprehensive early rehabilitation is both safe and effective.

In instances where adverse events are not invariably predictable, factors including patient characteristics, surgical procedures, indications, the type of physical therapy, and the timing of its initiation can be modulated and have a considerable impact on patient outcomes. This meta-analysis shows that early physical therapy does not present a significantly major risk of adverse events, both for lumbar discectomy and lumbar interbody fusion, when compared to the standard initiation of psychical therapy. These results can be highlighted by the relatively consistent direction of the outcomes shown among the included studies. Specifically, certain studies [15,23,26] suggested that complications were almost evenly distributed across the groups or slightly in favor of early physical therapy, yet never achieving a statistically significant difference. The dichotomous models in the meta-analysis indeed confirm this trend, both for lumbar discectomy and lumbar interbody fusion.

Post-physical therapy low back pain can represent a challenge for patients undergoing lumbar discectomy or lumbar intervertebral fusion procedures, manifesting with a highly variable incidence rate ranging from 3% to 43% [28,29]. Indeed, the surgical treatment involves subperiosteal decortication and the removal of bone segments from the vertebrae. This can induce muscular atrophy, weakness [30], diminished range of motion [31], and pain, leading to a fear of movement, stiffness, and an increased level of disability [32]. Hence, the impact on postoperative physical and mental health may extend beyond the initial expectations. Consequently, various rehabilitation programs have been developed to hasten symptom resolution, particularly pain, facilitate functional recovery and a return to employment, offer reassurance to patients, and, ultimately, prevent chronic pain, complications, and recurrences [33]. Our work reported discordant results in terms of low back pain after early physical therapy for lumbar interbody fusion, showing the need for more randomized control trials on this topic to provide clear guidance on the optimal timing for initiating physical therapy. Conversely, when considering early physical therapy after lumbar discectomy, there is moderate evidence that early physical therapy reduces pain at 12 weeks and 12 months postoperatively. These findings are consistent with those obtained by other reviews, which suggested that therapeutic programs should start between 4 and 6 weeks postoperatively [34]. 

### 4.1. Limitations of the Study

This work does not come without limitations. One is that we found quite consistent heterogeneity in the duration, intensity, type, and timing of initiation of the physical therapy interventions. The heterogeneity in the timing of initiation can potentially cause a significant limitation. However, the majority of the included studies [8,15,18,20,21,22,23,25,26] began within the first two postoperative weeks, and four [15,18,23,25] began within the first postoperative day, thereby reducing the impact of heterogeneity on our results. Nevertheless, it is imperative to exercise caution when acknowledging that the studies included in this review employed a diverse array of physical therapy protocols, extending from basic muscle strengthening exercises to sophisticated regimes incorporating both physical therapy and psychomotor education. 

Overall, the effectiveness of postoperative physical therapy in improving patient outcomes after lumbar surgery can greatly depend on the healthcare context of the region, highlighting the need for adaptable and resource-sensitive approaches in global spinal care. In developed countries, especially those with robust healthcare infrastructures close to metropolitan areas, postoperative rehabilitation programs are often comprehensive and standardized, featuring the latest in physiotherapeutic techniques and equipment. Patients in these areas typically have access to a range of specialists and multimodal therapies, which can lead to faster and more effective recovery outcomes.

Conversely, in developing countries or rural regions distant from large cities, physical therapy protocols post-surgery can be less consistent, with variations in the availability of specialized care and resources. Rehabilitation services might be more generic, less frequent, or rely heavily on patient self-management due to limited access to specialized healthcare facilities and professionals. This discrepancy can affect the speed and quality of recovery, potentially leading to longer periods of disability and higher rates of complications.

Despite this variability, a unifying philosophical approach was evident across all the included studies. Specifically, they conducted comparisons between cohorts that engaged in early initiation of any form of physical therapy and those patients who either commenced physical therapy at a later stage or did not participate in any physical therapy interventions. Another limitation is that only a small number of reported complications were reported by all the included studies, limiting the potential of picturing all adverse events that a patient can experience starting early physical therapy, particularly if these complications are unrelated to the surgical technique or spine exposure. However, the strength of this work is that it focused on clinically relevant parameters for patients, such as pain, function and disability, return to work, and possible complications, offering moderate evidence that early physical therapy could be beneficial for different aspects relevant to the patients.

### 4.2. Conclusions of the Study

This review indicates that employing early rehabilitation strategies for intervertebral disc disease could enhance results in terms of pain and disability without an enhanced risk of complications. However, it is evident there is a lack of randomized controlled trials (RCTs) on post-surgery rehabilitation for lumbar interbody fusion procedures, along with a significant diversity in the interventions proposed. There is a need for more research into the efficacy of integrative pre- and post-surgery rehabilitation programs to identify the most opportune moment to commence physical therapy following surgical procedures and understand the long-term impacts of these programs. Enhancing the quality of research in this area is crucial.

## Figures and Tables

**Table 1:** Details of the included studies; NS = non-specified; RMQ = Roland Morriss Questionnaire; DRI = Disability Rating Index; VAS = visual analogue scale.

Authors (Year)	Study Design (Level of Evidence)	Number of Patients (M:F)	Mean Age (±or Range or SD) (Years)	Surgery	Timing of PT	Intervention	Control	Outcomes	Complications
Kjellby-Wendt et al. (1998) [14]	Randomized controlled trial (I)	- Early active training program (EAT): 26 (18:8) - Control group: 26 (20:6)	- EAT group: 41 years (range, 24–68 years) - Control group: 39 years (range, 21–66 years)	Lumbar microdiscectomy	Postoperative day 1 in both groups, but control group had a less active program	Education, lumbar braces, stretching, muscular strengthening exercises	Patients in the control group were treated with a less active training program after lumbar discectomy (17 min shorter the first 6 weeks and 21 min shorter the last 6 weeks)	Patients rehabilitated according to the EAT program had significantly less intense pain and more range of motion in the lumbar spine at 12 weeks after surgery. One year after surgery, there was no significant difference between groups in duration of sick leave and treatment satisfaction, even though the patients in the early therapy group were more satisfied compared to the controls (88% vs. 67%)	- ETA: 1 reoperation, 1 spondylolisthesis - Control: 1 reoperation, 1 reoperation at the same level of the lumbar spine
Kjellby-Wendt et al. (2001) [15]	Prospective randomized study (II)	- Early treatment group (EAT): 26 (18:8) - Control: 24 (18:6)	- Treatment: 41 (24–68) - Control: 37 (26–66)	Lumbar microdiscectomy	Postoperative day 1 in both groups, but control group had a less active program	Exercises to restore mobility of the trunk, reduce local edema, and stretch legs. Patients were encouraged to swim or jog	Traditional, less active training program	Both groups improved pain severity and state of anxiety. The multidimensional pain inventory improved more in the EAT group	NS
Erdogmus et al. (2007) [16]	Randomized control trial (I)	- No therapy: 40 (25:15) - Sham therapy: 40 (21:19) - Physical therapy: 40 (21:19)	- No therapy: 41.8 ± 10.4 - Sham therapy: 42.3 ± 9.8 - Physical therapy: 39.8 ± 10.5	Standard laminectomy and either discectomy or microdiscectomy procedure	Within the first postoperative week in both groups, but control group had sham therapy or no therapy at all until after the first 3 months	Education, stretching, endurance exercises	- No therapies for the first 3 postoperative months. - Sham therapy: only massages for 30 min	After 12 weeks, low back pain was significantly less in physical therapy group than in untreated group. After 1.5 years, there were no significant outcome differences, including secondary outcomes (return to work, patient satisfaction, and activity of daily living)	- No therapy: 2 re-herniations - Sham therapy: 2 re-herniations - Physical therapy: 1 re-herniation
Millisdotter et al. (2007) [8]	Prospective controlled trial, not randomized—Level II	- Total: 56 (36:20) - Early Training (ET): 25 (19:6) - Control: 31 (17:14)	- Total: 38 - ETG: 37 - CG: 31	Open microscopic lumbar disc discectomy	- Early training group (ETG): 2 weeks after surgery - Control group (CG—traditional training): after 6 weeks	Education, neuromuscular closed-chain exercises	Control group (CG—traditional training): stabilization exercises mainly using different types of stationary gym equipment and focused on coordination and mobility	Early neuromuscular customized training had a superior effect on disability (RMQ and DRI), with a significant difference compared to traditional training at follow-up 12 months after surgery. No differences in terms of pain (VAS) between groups	- Early training: 2 revision surgeries - Control: 1 revision surgery
Newsome et al. (2009) [17]	Prospective randomized control trial (I)	- Intervention group: 15 (7:8) - Control: 15 (11:4)	- Intervention group: 38 (27–43.3) - Control: 37 (30.5–45)	Lumbar microdiscectomy	- Intervention group: postoperative hour 2 - Control: postoperative day 1	Passive hip and knee flexion toward the chest. Mobilization out of bed and education	Started on postoperative day 1, similar care but no knee and hip flexion	Significantly reduced time to independent mobility and return to work (median 6 vs. 8 weeks in the intervention group compared with the control group). At 15 h after surgery, independent mobility was attained in 80 and 40% of the intervention and control groups, respectively. There were no significant differences in disability and pain scores at 4 weeks and 3 months	- Intervention: 1 recurrence of symptoms - Control: 1 recurrence of symptoms
Abbott et al. (2010) [18]	Randomized control trial (I)	- Total: 107 (41:66) - Control: 54 (23:31) - Physical therapy: 53 (18:35)	- Control: 50.3 ± 10 - Physical therapy: 51.0 ± 10.9	Lumbar fusion surgery	Starting from postop week 3 in both groups, but controls had a less active program	Education, motor relearning exercises, cognitive behavioral relearning exercises	Patients in the control group received education on walking, daily living exercises, and activity restrictions	Physical therapy improved functional disability, self-efficacy, outcome expectancy, and fear of movement/(re)injury significantly more than control group at the respective follow-up occasions. Similar results occurred for pain coping, but group differences were non-significant at 2 to 3 years of follow-up	- Control: 2 removals of instrumentation, 1 adjacent-level degeneration, 2 pseudoarthroses - Physical therapy: 5 removals of instrumentation, 2 adjacent-level degenerations, 5 pseudoarthroses
Oestergaard et al. (2012) [9]	Randomized control trial (II)	- Total: 82 (38:44) - Intervention: 41 (21:20) - Control: 41 (17:24)	- 6 weeks: 52 ± 8.5 - 12 weeks: 51.3 ± 9.9	Lumbar spine fusion for degenerative disc disease	- Intervention: starting at postop week 6 - Control: postop week 12	Education, muscle strengthening, exercises focusing on trunk and large muscle groups	The same physical therapy protocol, but started at 12 weeks	According to the Oswestry Disability Index, at 1-year follow-up, the 6-week group had significantly lower median reduction compared to the 12-week group. The Dallas Pain Questionnaire showed the same tendency overall, and daily activities were significantly reduced in favor of the 12-week group. For back pain, the 6-week group had a median reduction similar to the 12-week group. The results at 6 months of follow-up were similar. No difference was found in return to work 1 year post-surgery	- 6 weeks: 3 revision surgeries, 2 removals of instrumentation - 12 weeks: 5 revision surgeries, 2 removals of instrumentation
Ju et al. (2012) [19]	Randomized control trial (II)	- Exercise therapy group (ETG): 7 - Control: 7	- ETG: 45.2 ± 3.96 - CONG: 46.2 ± 5.3	Lumbar disc discectomy	- Exercise Therapy Group: postoperative week 2 - Control: no physical therapy	Lumbar extension program, resistance exercises	The control group did not participate in any exercise rehabilitation programs	ETG showed significant improvements in all items that measured lumbar extensor muscle strength and pain after the intervention, but the control group did not exhibit any significant improvements	NS
Oestergaard et al. (2013) [20]	Randomized control trial (I)	- Total: 82 (38:44) - 6 weeks: 41 (21:20) - 12 weeks: 41 (17:24)	- 6 weeks: 52 ± 8.5 - 12 weeks: 51.3 ± 9.9	Lumbar spine fusion for degenerative disc disease	- Intervention: starting at postop week 6 - Control: postop week 12	Education, muscle strengthening, exercises focusing on trunk and large muscle groups	The same physical therapy protocol, but started at 12 weeks	No statistically significant difference was found in walking distance or fitness over time. In both groups, the patients achieved an overall increase in walking distance but no improvement in fitness	- 6 weeks: 3 revision surgeries, 2 removals of instrumentation - 12 weeks: 5 revision surgeries, 2 removals of instrumentation
Oestergaard et al. (2013) [21]	Randomized control trial (I)	- Total: 82 (38:44) - 6 weeks: 41 (21:20) - 12 weeks: 41 (17:24)	- 6 weeks: 52 ± 8.5 - 12 weeks: 51.3 ± 9.9	Lumbar spine fusion for degenerative disc disease	- Intervention: starting at postop week 6 - Control: postop week 12	Education, muscle strengthening, exercises focusing on trunk and large muscle groups	The same physical therapy protocol, but started at 12 weeks	The 6w group had significantly poorer outcome in relation to functional disability than the 12w group. The same tendency was found for QALY, although this difference was not statistically significant	- 6 weeks: 2 readmissions before rehabilitation - 12 weeks: 1 readmission before rehabilitation
Ozkara et al. (2015) [22]	Prospective randomized control study (II)	- Treatment group: 15 (6:9) - Control: 15 (7:8)	- Treatment group: 48.5 ± 11.9 - Control: 44.1 ± 8.8	Lumbar disc discectomy	Postoperative day 1 in both groups, but control group did not perform any exercises but only education	Education, exercises for pelvic tilt, abdominal and isometric quadriceps strengthening. Back exercises, leg raises, and hip flexions were added after the sixth week	Only instructions regarding lying, standing, sitting, and walking	When the groups were compared at week 12, a statistically significant difference was found in the VAS, Oswestry Low Back Pain Disability Questionnaire, and physical functioning of the SF-36, including body pain and social functioning subparameters. There was no significant difference in terms of return to work and patient satisfaction	NS
Oosterhuis et al. (2017) [23]	Multicenter, randomized, controlled trial (I)	- Experimental group: 92 (38:54) - Control: 77 (20:57)	- Exp: 47 (12) - Con: 47 (12)	Lumbar disc discectomy	- Experimental group: starting the first week after discharge - Control: only education after discharge	Education, daily activity training, gradually increasing intensity of exercises	Participants assigned to the control group were not referred for rehabilitation after discharge from the hospital	No clinically relevant or statistically significant overall mean differences between rehabilitation and control for any outcome adjusted for baseline characteristics (global perceived recovery, functional status, leg pain, back pain, physical health, and mental health)	- Exp: 1 nerve root injury, 2 dural tears - Control: 1 nerve root injury, 2 dural tears, 1 increase in sensimotor deficit
Kernc et al. (2018) [24]	Randomized controlled trial (I)	- Control group: 14 (5:9) - Training group: 13 (9:4)	- Control group: 60.3 ± 8.1 - Training group: 61.1 ± 8	One-level instrumented trans-foraminal interbody fusion	- Training group: starting 3 weeks after the surgery - Control group: started 3 months postoperatively	Isometric exercises focused on trunk extension, flexion, and lateral flexion muscles. Leg adduction and hip extension	- Control group: no exercises or physical therapy prior to 3 months postoperatively	Both groups improved their walking speed after 3 months, although improvement in the training group was significantly greater than that in the control group. The training group significantly improved in all isometric trunk muscle measurements	- Training group: 0 - Control group: 2 hardware loosenings
Zhang et al. (2021) [25]	Randomized controlled trial (I)	- Total: 92 (48:44) - Intervention group: 46 - Control group: 46	57.4 ± 6.1 (20–68)	Percutaneous trans-foraminal endoscopic discectomy	- Intervention group: started at postoperative day 1 - Control group: no exercises were performed	Education, daily activity training, extension and flexion exercises of lower limbs, back muscle exercises	Control group performed routine functional exercises after their operations (not mentioned when)	Scores for residual lumbocrural pain, straight leg raising, muscle strength, sensory (skin), nerve reflex, and lumbar function of patients in the intervention group were significantly better than those of the control group	NS

## Data Availability

Data are available, with adequate reason, upon request from the corresponding author.
